# Implementation of a youth‐adult partnership model in youth mental health systems research: Challenges and successes

**DOI:** 10.1111/hex.12554

**Published:** 2017-03-14

**Authors:** Olivia S. Heffernan, Tyson M. Herzog, Jordana E. Schiralli, Lisa D. Hawke, Gloria Chaim, Joanna L. Henderson

**Affiliations:** ^1^ Centre for Addiction and Mental Health Toronto ON Canada; ^2^ University of Toronto Toronto ON Canada

**Keywords:** mental health systems change, patient engagement, youth‐adult partnership

## Abstract

**Background:**

By integrating Youth–Adult Partnerships (Y‐APs) in organizational decision making and programming in health‐care settings, youth can be engaged in decisions that affect them in a way that draws on their unique skills and expertise. Despite challenges, Y‐APs can have many benefits for youth and adults alike, as well as for the programmes and initiatives that they undertake together.

**Objective:**

This article describes the development, implementation and success of a Y‐AP initiative at the McCain Centre at the Centre for Addiction and Mental Health, a large urban hospital.

**Method:**

The McCain Y‐AP implementation model was developed based on the existing literature, guided by the team's progressive experience. The development and implementation procedure is described, with indicators of the model's success and recommendations for organizations interested integrating youth engagement.

**Results:**

The McCain Y‐AP has integrated youth into a wide range of mental health and substance use‐related initiatives, including research projects, conferences and educational presentations. The model of youth engagement is flexible to include varying degrees of involvement, allowing youth to contribute in ways that fit their availability, interest and skills. Youth satisfaction has been strong and both the youth and adult partners have learned from the experience.

**Discussion:**

Through the McCain Y‐AP initiative, youth engagement has helped advance numerous initiatives in a variety of ways. Flexible engagement, multifaceted mentorship, reciprocal learning and authentic decision making have led to a successful partnership that has provided opportunities for growth for all those involved. Health‐care organizations interested in engaging youth can learn from the McCain Y‐AP experience to guide their engagement initiatives and maximize success.

## 1 Introduction

Youth voices are imperative to inform decision making and programming that directly impact youth. Collaborative relationships in which youth can interact with adults and engage in shared decision making and programming are referred to as youth–adult partnerships (Y‐APs).^1^ Through Y‐APs, youth gain skills for positive and healthy development, leading to improved social trust, school achievement^2^ and lifelong community citizenship.^3^ Moreover, youth gain awareness, new skill sets and control over their own health leading to significantly better health outcomes.^3^ Y‐APs can manifest in several contexts, including civic engagement,^4^ community programmes,^5^, ^6^ education^7^, ^8^ and research.^9^ There have been recent efforts to introduce Y‐APs into the mental health‐care system,^10^, ^11^ however, research remains limited. Fostering an open environment where youth can contribute to planning can increase the effectiveness of the system, and also protective factors for the youth.^12^

Y‐APs have been successfully incorporated into the working structure of a research and innovation team within the McCain Centre at the Centre for Addiction and Mental Health (CAMH), from co‐facilitating workshops to being leading authors of this paper. The team facilitates engagement of youth with lived experience of mental health challenges with mental health service providers and researchers to inform service planning, policy development and research. Accordingly, we review the key components of Y‐APs drawn from the literature, highlight how they are incorporated into the McCain team, and discuss challenges and successes.

### 1.1 Youth‐adult partnerships

Creating Y‐APs can be challenging, especially in settings where power imbalances are expected, such as in a school environment, or research teams where youth are often seen as consumers rather than stakeholders.^13^ Accordingly, implementing successful Y‐APs requires flexibility, mentorship, authentic decision making and reciprocal learning.^2^, ^8^, ^11^ Flexibility involves malleable roles that allow both youth and adults to showcase their skills and talents. In addition, flexibility regarding deadlines, milestones and overall goals is critical.^11^ Moreover, to achieve their goals, Y‐APs must not replicate the power imbalance seen in parent–child or student–teacher relationships^15^ but should create safe, welcoming environments where all parties' opinions and contributions are respected and valued.^16^ To recognize that each person has a unique skill set, all partners must be willing to abandon preconceived notions of the way partnerships or relationships between youth and adults work. Empowerment is not solely the responsibility of the adults or the youth, but is rather a shared duty involving co‐learning^16^, defined as reciprocal learning.

In Y‐APs, youth are decision makers rather than consumers or consultants.^8^ That is, youth have responsibilities and are trusted to make decisions that directly influence team projects and outcomes in their own lives. Wong discusses possible degrees of participation using Hart's model of youth engagement.^16^ The model is an eight‐rung ladder depicting progression from poor to excellent engagement typologies. At the *non‐participation* level, youth are involved only to have youth engagement appear important, but are not provided with an understanding of the issues nor decision‐making opportunities. Progressing up the ladder, youth engagement becomes both more fulsome and authentic. Youth transition from understanding projects and having their views respected at the lowest level of *participation* to having complete liberty over projects, including using their own ideas and inviting adults to join them in decision making at the highest level.

The nature of the relationship in Y‐APs is one of reciprocal learning: all parties are both teachers and learners, relying on individual strengths,^9^ as opposed to traditional structures of age and education determining power. For example, in research, adults may be experts in research methods, while youth may be experts about their school environment. Together, diverse expertise works in cohesion to create a successful, well‐grounded research project.

### 1.2 McCain Y‐AP structure

The McCain Y‐AP's structure is composed of two youth with lived experience of mental health issues (co‐authors O.H. and T.H.) and two non‐youth mental health professionals (G.C. and J.H.; “adults”). The youth are full‐time team members at CAMH, employed as youth engagement facilitators (YEFs), contributing project ideas and engaging youth. The YEFs and adults co‐founded the National Youth Advisory Committee (NYAC), a group of young Canadians aged 12‐29 who are passionate about mental health and substance abuse issues. YEFs guide NYAC members in creating and implementing youth‐led mental health projects. YEFs provide NYAC members opportunities to contribute to research within and outside of McCain through advisory groups, consultations and other mechanisms.

## 2 **McCain Model of Youth Engagement**

**Figure 1:**
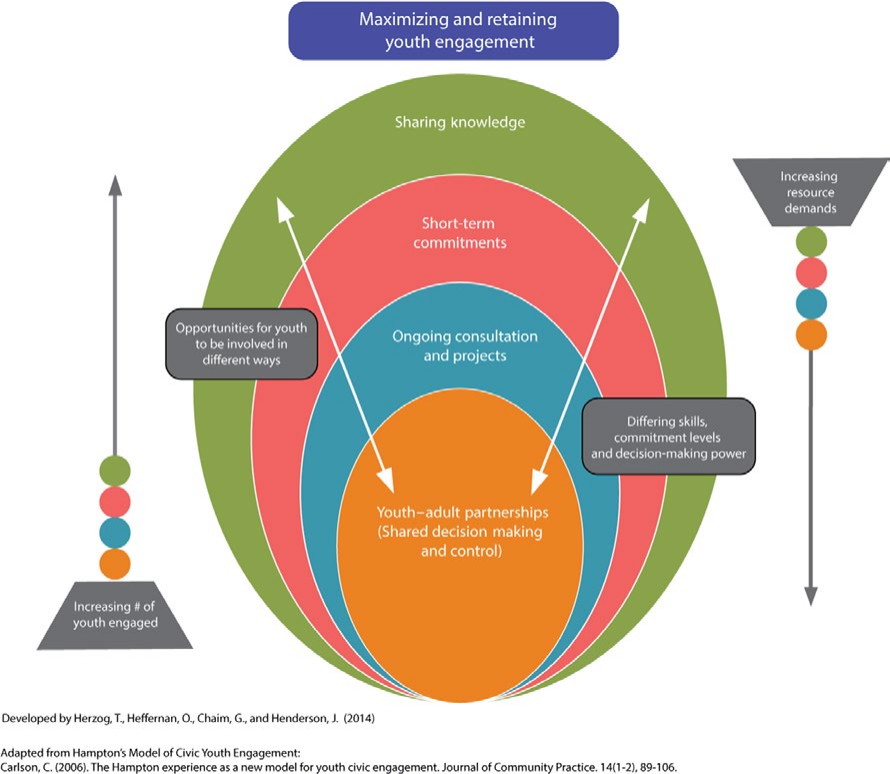
Model of youth engagement

Based on the Hampton Model of Youth Civic Engagement^4^ and the TYPE Pyramid,^16^ the McCain Centre has developed the McCain Model of Youth Engagement (see Figure 1). This model was developed through: 1) literature review of Y‐APs, other forms of youth engagement and models of participatory research; 2) discussing Y‐AP goals for youth engagement; and 3) trial‐and‐error in engaging young people. The Model provides other organizations with a conceptual framework to guide their youth engagement strategies. Each of the engagement methods provides different involvement opportunities for youth. Youth are able to use different skills, make large or small commitments and exercise their decision‐making power.

### 2.1 Key attributes of the model

#### 2.1.1 Flexibility

Individuals with mental illness may have different working styles and needs than the general working public.^17^ Situated within a hospital setting with unionized positions and established policies, the McCain Y‐AP was challenged to problem solve to balance accommodating YEFs' needs and hospital structures. For example, regarding work hours, the team adopted a flexible approach, trusting YEFs to determine their own schedules with manager approval. These steps addressed YEFs' unique needs, and were also important to the task of engaging youth most often available evenings and weekends. In addition, YEFs were each assigned workstations with fluorescent overhead lighting, with minimal natural light. Due to concerns about possible effects on headaches and mental health, especially during Canada's dark winter months, YEFs used light therapy lamps at their desks and were permitted daily use of nearby meeting rooms with large windows. Because the McCain YEF roles were initially loosely defined, the team was able to work together to co‐design and structure roles and deliverables around individual skills and interests, enabling YEFs to develop skills they considered relevant to their future aspirations, setting the groundwork for mentorship.

NYAC exemplifies flexibility through the committee's design, structure and interactions. After reviewing background material, interested youth can become members by completing an online survey regarding basic personal information; disability accommodations; technology access; preferred communication modes; current interests and talents; and talents they wish to develop through NYAC involvement. This information is used to guide committee processes and match members with relevant, personally meaningful opportunities.

#### 2.1.2 Mentorship

In line with the literature,^15^ our initiative included both formal and informal mentorship. On an on‐going basis McCain adults provided mentorship about research, policy and professional development. Also, through funding from the Margaret and Wallace McCain Centre for Child, Youth & Family Mental Health at CAMH, YEFs are conducting a youth‐led research project with formal mentorship from a co‐author (JH). NYAC contributes to the research project under the mentorship of YEFs. NYAC members are contributing to every project stage, including creation, implementation, evaluation, interpretation and reporting. Notably, it is important to be intentional about mentorship relationships: unintentional changes, especially without appropriate preparation, can compromise unique Y–AP mentorship relationships and upset the power balance with resulting dysfunction in the Y–AP relationship.

McCain managers/supervisors have also provided mentorship by supporting YEFs in developing new skills and building relationships. For example, YEFs expressed interest in graphic design and were then provided with software, time and opportunities to develop these transferable skills. They can then enhance research team presentations with professional‐looking charts and graphics and collaborate with NYAC members to create project visuals. Similarly, YEFs and managers/supervisors attend many conferences and events together that afford opportunities for networking. Prior to these events, meetings are held to discuss questions or concerns, decide which initiatives to promote to other organizations, and choose key messages.

Regarding personal qualities and skills YEFs should have for effective mentorship,^2^ YEFs are expected to be non‐judgmental, passionate, relatable, well‐organized young adults who are positive communicators and active listeners. Hired for these characteristics, YEFs strive to continually use these skills and emulate these qualities in their interactions with NYAC members as a form of informal mentorship and role modeling.

Also to mentor NYAC members, YEFs gather information, match projects to member skills and interests, provide opportunities outside of NYAC, facilitate networking between NYAC members and organizational bodies, and conduct on‐going check‐ins using a communication mode of each member's choice (eg, in‐person meetings, email, telephone call, conference call). NYAC mentorship also occurs via an online private working group to share ideas, form connections, and seek assistance from other members with particular skills.

#### 2.1.3 Authentic decision making

Authentic decision making, an essential component of Y‐APs, must be developed intentionally to ensure full implementation and maintenance. Y‐APs can slide into “tokenism” “unless genuine opportunities exist for youth to participate in decision‐making processes” (p. 222).^18^ In the McCain Y‐AP, authentic youth engagement is clearly demonstrated through active participation in research, proposal writing, presentation development and facilitation and by being recognized as equal team members.

Authentic decision‐making opportunities were built into NYAC including on‐going open dialogue with members about NYAC structure and operations which has resulted in recommendations that have been implemented. For example, in response to member concerns that NYAC's webpage was overwhelming and cluttered, YEFs implemented several solutions, including using another platform, categorizing topics and creating a page strictly for member opportunities, changes that were well received by members. In addition, NYAC members have opportunities to co‐create their own projects, with YEFs providing guidance and structure but with ultimate decision making by NYAC youth. NYAC's first antistigma campaign, *#selfree*, provides a strong example of this approach.

#### 2.1.4 Reciprocal learning

Reciprocal learning is an important Y‐AP component that can facilitate personal and/or professional growth. Reciprocal learning occurs when each party is considered an expert in an area and able to teach the other members, regardless of age or “power.” Both youth and adults are “teachers” and “students” in different areas. The McCain YAP upholds this value. Among the constant learning opportunities for YEFs, co‐authors GC and JH provide context and guidance on navigating oftentimes confusing organizational structures, in addition to practical skills in proposal writing and research. As teachers, YEFs show adult partners how to engage youth more effectively, effective technology use, and strategies for enhancing “youth‐friendliness.”

In NYAC, reciprocal learning occurs between members, YEFs and adult partners. Viewing everyone as having expertise, NYAC members have collaborated with YEFs to create webinars on topics they feel comfortable teaching to others and broadcasting to NYAC members. In addition, through a private, invitation‐only online working group for focused discussion of projects and related topics, members share thought‐provoking articles or examples of antistigma campaigns, providing substantial learning opportunities. Similarly, an “Opportunities Page” allows NYAC members and YEFs to post options for involvement in mental health and substance use initiatives, allowing members to share their knowledge and creating a feeling of community.

### 2.2 Success of the model

The McCain Model of Youth Engagement has provided a successful framework for engaging young people in a health‐care research and advocacy setting. Potential measures of its success arose organically and include the frequency of requests for youth collaboration and consultations, the breadth and scope of their engagement and member satisfaction.

The high demand for the Y‐AP YEFs' collaboration and consultation is an indicator of the program's success. The rate of invitations for YEF involvement in research, policy, front‐line and systems‐level initiatives required the development of new policies outlining the parameters of YEF involvement. Capitalizing on this demand, YEFs were able to connect NYAC members with those requesting the youth voice, thus creating more networking opportunities for youth.

The initiative's success is also evident through high levels of sustained, diverse youth engagement in NYAC. This is demonstrated in various ways, including successful social media campaigns, the diversity of the youth involved, an active online working group and the number of successful connections made between members and various local, provincial and national engagement opportunities. The diversity and enthusiasm of the NYAC group, the successfully developed youth‐led projects, the high demand for collaboration and the anecdotal and observational indicators show that the McCain Model of Youth Engagement was implemented with success.

## 3 Discussion

The McCain Model of Youth Engagement at CAMH was developed using the best available evidence to support the creation of a Y‐AP for a mental health‐care environment and bolster youth participation in research and other processes. Through collaborative implementation that emphasizes framework‐informed guiding principles and adaptation, the Y‐AP grows increasingly better equipped to meet the needs of the YEFs and the youth they engage. By upholding core principles of flexibility, mentorship, authentic decision making and reciprocal learning, the McCain Y‐AP fosters healthy relationships and genuine partnerships. Within two years, the Y‐AP has built McCain capacity to meaningfully engage youth in a wide variety of activities, including research projects, educational presentations, conferences and media and promotional activities.

CAMH's NYAC emerged from the McCain Model of Youth Engagement. NYAC's approach to engagement is flexible, casual, developmentally‐informed and responsive to individual needs. NYAC is a low‐barrier option that allows young Canadians to get involved in mental health conversations according to their skills and availability. Implementation of this model has resulted in high levels of meaningful youth engagement.

The McCain Y‐AP integrates key components of youth engagement: flexibility, mentorship, authentic decision making and reciprocal learning,^2^, ^11^, ^16^ attaining the highest rung of Hart's (1992) youth engagement ladder, ie, youth‐initiated, shared decisions with adults. The challenges of power imbalances^14^ have been successfully navigated and youth have been integrated into the McCain team.

Learning from the challenges previously reported,^11^ flexibility was integrated into the model, enhancing its success. A multi‐level mentorship structure, in which adults mentored YEFs, adults learned from YEFs and YEFs mentored a broader network of youth, created a welcoming environment where a diversity of opinions, contributions and strengths are respected and valued.^9^, ^16^ The result has been skill building that prepares youth to become engaged adults, with potential positive long‐term impacts on their health outcomes,^3^, ^12^ while helping guide the adult allies in the development of youth‐friendly initiatives.

### 3.1 Implications/Recommendations

This process has been a considerable learning experience for the McCain Y‐AP team and the organization as whole. Based on this experience, we propose several recommendations for health‐care organizations wishing to implement Y‐AP initiatives:

- Develop partnerships with young people early in the planning process.
- Be proactive about planning and developing guidelines for youth engagement, recognizing potential organizational barriers and/or policies that may need to be revised.
- Recognize the need for training about youth–adult partnership and co‐creation.
- Establish a method of recruitment that speaks to the diversity of the individuals being sought.
- Develop an understanding of the needs of the organization and/or projects to guide engagement strategies.

Y‐AP planners should remember that engagement and partnership do not have to be perfect. The key is to stay true to core values and strategies in finding creative ways to reach out to youth. As illustrated by the McCain Model of Youth Engagement, there are many ways to engage youth appropriately and meaningfully, even without extensive resources.

### 3.2 Limitations

This paper describes the McCain experience, which may not generalize to environments where broad online contributions and facilitation are not feasible. NYAC's membership may have been bolstered by CAMH's outstanding academic reputation; the McCain Model may be more difficult to implement without the support of a highly regarded organization. On‐going research and implementation of Y‐APs is needed to further explore the factors necessary to success, as well as factors that may be adaptable to the local context.

## 4 **Conclusions**

This paper describes the McCain Model of Youth Engagement and youth–adult partnerships, illustrating an effective process for engaging youth in mental health and addiction research, planning and system building. Through flexible engagement and mentorship, the McCain team has created an environment of reciprocal learning and authentic shared decision making that has benefitted youth and adults alike. The team's research projects, presentations, conferences and media visibility have been enhanced and made more accessible to youth as the Y‐AP has grown into an evidence‐informed partnership that draws on the unique skills and knowledge of all those involved. Health‐care organizations interested in engaging youth can learn from the McCain Y‐AP experience to guide their engagement initiatives and maximize success.

## Conflicts of Interests

None.

## References

[1] Zeldin S , Camino L , Mook C . The adoption of innovation in youth organizations: creating the conditions for youth‐adult partnerships. J Community Psychol. 2005;33:121‐135.

[2] Zeldin S , Christens BD , Powers JL . The psychology and practice of youth‐adult partnership: bridging generations for youth development and community change. Am J Community Psychol. 2013;51:385‐397. 23054170 10.1007/s10464-012-9558-y

[3] Suleiman AB , Soleimanpour S , London J . Youth action for health through youth‐led research. J Community Pract. 2006;14:125‐145.

[4] Carlson C . The Hampton experience as a new model for youth civic engagement. J Community Pract. 2006;14:89‐106.

[5] Jones KR , Perkins DF . Youth and adult perceptions of their relationships within community‐based youth programs. Youth Soc. 2006;38:90‐109.

[6] Libby M , Rosen M , Sedonaen M . Building youth‐adult partnerships for community change: lessons from the Youth Leadership Institute. J Community Psychol. 2005;33:111‐120.

[7] Joselowsky F . Youth engagement, high school reform, and improved learning outcomes: building systemic approaches for youth engagement. NASSP Bull. 2007;91:257‐276.

[8] Mitra DL . Collaborating with students: building youth‐adult partnerships in schools. Am J Educ. 2009;115:407‐436.

[9] Kirshner B , O'Donoghue J , McLaughlin M . Youth‐adult research collaborations: bringing youth voice to the research process In: L. Mahoney J, W. Larson R, S. Eccles J, eds. Organized Activities as Contexts of Development Extracurricular Activities, After‐School and Community Programs. Mahwah, NJ: Lawrence Erlbaum Associates, 2005:131‐156.

[10] Davidson M , Manion I , Davidson S , Brandon S . For youth by youth: innovative mental health promotion at Youth Net/Réseau Ado. Vulnerable Children and Youth Studies. 2006;1:269–273. doi:10.1080/17450120601010171.

[11] Ramey HL , Rose‐Krasnor L . The new mentality: youth‐adult partnerships in community mental health promotion. Child Youth Serv Rev. 2015;50:28‐37.

[12] Norman J . Building effective youth‐adult partnerships. Transitions: The rights. Respect. Responsibility. Campaign. 2001;14:10‐12 & 18.

[13] Mitra DL . Student voice or empowerment? Examining the role of school‐based youth‐adult partnerships as an avenue toward focusing on social justice. Int Electronic J Leadership Learn. 2006;10.

[14] Mitra DL . Strengthening student voice initiatives in high schools: an examination of the supports needed for school‐based youth‐adult partnerships. Youth Soc. 2009;40:311‐335.

[15] Camino LA . Youth‐adult partnerships: entering new territory in community work and research. Appl Dev Sci. 2000;4:11‐20.

[16] Wong NT , Zimmerman MA , Parker EA . A typology of youth participation and empowerment for child and adolescent health promotion. Am J Community Psychol. 2010;46:100‐114. 20549334 10.1007/s10464-010-9330-0

[17] MacDonald‐Wilson KL , Rogers ES , Massaro JM , Lyass A , Crean T . An investigation of reasonable workplace accommodations for people with psychiatric disabilities: quantitative findings from a multi‐site study. Community Ment Health J. 2002;38:35‐50. 11892855 10.1023/a:1013955830779

[18] Scheve JA , Perkins DF , Mincemoyer C . Collaborative teams for youth engagement. J Community Pract. 2006;14:219‐234. PMC3297413 22408761

